# A robust classifier of high predictive value to identify good prognosis patients in ER-negative breast cancer

**DOI:** 10.1186/bcr2138

**Published:** 2008-08-28

**Authors:** Andrew E Teschendorff, Carlos Caldas

**Affiliations:** 1 Breast Cancer Functional Genomics Laboratory, Cancer Research UK Cambridge Research Institute, Cambridge, CB2 0RE, UK.; 2 Department of Oncology University of Cambridge, Li Ka-Shing Centre, Robinson Way, Cambridge CB2 0RE, UK.; 3 Cambridge Breast Unit, Addenbrookes Hospital, Cambridge University Hospitals NHS Foundation Trust, Hills Road, Cambridge, UK.

## Abstract

**Introduction:**

Patients with primary operable oestrogen receptor (ER) negative (-) breast cancer account for about 30% of all cases and generally have a worse prognosis than ER-positive (+) patients. Nevertheless, a significant proportion of ER- cases have favourable outcomes and could potentially benefit from a less aggressive course of therapy. However, identification of such patients with a good prognosis remains difficult and at present is only possible through examining histopathological factors.

**Methods:**

Building on a previously identified seven-gene prognostic immune response module for ER- breast cancer, we developed a novel statistical tool based on Mixture Discriminant Analysis in order to build a classifier that could accurately identify ER- patients with a good prognosis.

**Results:**

We report the construction of a seven-gene expression classifier that accurately predicts, across a training cohort of 183 ER- tumours and six independent test cohorts (a total of 469 ER- tumours), ER- patients of good prognosis (in test sets, average predictive value = 94% [range 85 to 100%], average hazard ratio = 0.15 [range 0.07 to 0.36] p < 0.000001) independently of lymph node status and treatment.

**Conclusions:**

This seven-gene classifier could be used in a polymerase chain reaction-based clinical assay to identify ER- patients with a good prognosis, who may therefore benefit from less aggressive treatment regimens.

## Introduction

Oestrogen receptor (ER) negative (-) breast cancer accounts for about 30% of all breast cancer cases and generally has a worse prognosis compared with ER positive (+)disease [1,2]. Nevertheless, a significant proportion of ER- cases have shown a favourable outcome and could potentially benefit from a less aggressive course of therapy [3]. Reliable identification of such ER- patients with a good prognosis is, however, difficult and at present only possible through examining histopathological factors.

Recently, attempts have been made to explain the observed clinical heterogeneity of ER- disease in terms of gene expression signatures [4-7]. However, most of these studies clearly indicated the difficulty of identifying a prognostic gene expression signature for ER- disease [4,6,7], unlike ER+ breast cancer where a multitude of alternative prognostic signatures have been identified [3,8-11]. Nevertheless, using an integrative analysis of gene expression microarray data from three untreated (no chemotherapy) ER- breast cancer cohorts (a total of 186 patients) [3,8,10] and a novel feature selection method [11], it was possible to identify a seven-gene immune response expression module associated with good prognosis,. This suggests that at least part of the observed clinical heterogeneity in ER- disease can be explained on the basis of mRNA expression levels [5]. Specifically, overexpression of this immune response gene module identified a subclass of basal ER- breast cancer, about 25% of all ER- cases, with a reduced risk of distant metastasis (Hazard ratio [HR] = 0.49; range 0.29 to 0.83; p = 0.009) compared with ER- cases without overexpression of this module [5], a result that was validated in two independent untreated test cohorts (58 ER- samples) [9,12].

The important role that immune system-related gene expression signatures play in breast cancer prognosis has been further supported by four recent reports [13-16]. Specifically, one study reported that high expression of lymphocyte-associated genes identifies a good prognosis subgroup within lymph node negative (LN-) human epidermal growth factor receptor 2 positive (HER2+) breast cancer [13]. A further study focused on LN- breast cancer and identified a prognostic B-cell metagene signature, confirming that overexpression of this signature correlated with good prognosis in ER- breast cancer, while underexpression correlated with good prognosis in ER+ breast cancer [14]. A similar contrasting result between ER- and ER+ breast cancer was also found by deriving a gene expression signature for lymphocytic infiltration (LI) and demonstrating its positive and negative association with good prognosis in ER- and ER+ disease, respectively [15]. All these results are consistent with our findings and highlight the importance of stratifying breast cancer patients into ER+ and ER- subtypes before associations with clinical outcome can be derived [5,16].

The discovery and construction of a molecular classifier that can robustly identify ER- patients with a good prognosis is important for two main reasons. First, identification of ER- patients with a good prognosis based on histopathological predictors like LN status or Adjuvant! is far from optimal [17]. Second, reliable identification of ER- patients of good prognosis could help guide the management of ER- patients further, by providing less aggressive treatment regimens for such patients. Building on our previous results [5] here we report on the construction of a seven-gene prognostic classifier and further validate this single-sample predictor across six (four untreated and two partially treated) independent ER- breast cancer cohorts: 'UPP' [12], 'JRH-2' [9], 'UNC248' [18], 'CAL' [19], 'Loi' [20] and 'Kreike' [6]. This therefore confirms the validity of this classifier in more than 469 ER- patients.

## Materials and methods

### Linear and quadratic discriminant analysis

Before discussing Mixture Discriminant Analysis (MDA), it is convenient to briefly review Linear Discriminant Analysis (LDA) and Quadratic Discriminant Analysis (QDA) [21]. We assume that we have a training data set *X *of dimension *p *× *N*, where *p *is the number of dimensions (ie, genes) and *N *is the number of training samples (ie, tumour samples). We also assume that we have a test set *Y *of dimension *p *× *n *and that we have *C *phenotype classes among the training set samples.

In the training process of discriminant analysis one attempts to learn parameters that specify the clusters associated with each of the phenotype classes. In the maximum likelihood framework, one learns parameters (*π*, *θ*) = (*π*_*k*_, *θ*_*k *_= 1,..., *C*) such that the likelihood function

(1) $$L(\pi ,\theta )=p(X|\pi ,\theta )=\prod_{i=1}^{N}\sum_{k=1}^{C}\pi_{k}f_{k}(x_{i}|\theta_{k})$$

is maximised. In the above, *f*_*k *_denotes the probability function that specifies the probability that the observation *x*_*i *_is generated from cluster *k*, *π*_*k *_denotes the weight of this cluster and *θ*_*k *_parameterises the cluster. The optimisation of the likelihood is performed using the EM-algorithm, subject to the constraint that $\sum_{k=1}^{C}\pi_{k}=1$, yielding estimates $\left(\hat{\pi },\hat{\theta }\right)$.

Having estimated the parameters, we can now classify a test sample *y *using Bayes' Theorem as follows. The probability that *y *belongs to class *k *is just the posterior probability *p*(*k*|*y*), which by Bayes' Theorem can be written as

(2) $$p(k|y)=\frac{{\hat{\pi }}_{k}f_{k}(y|{\hat{\theta }}_{k})}{\sum_{c=1}^{C}{\hat{\pi }}_{c}f_{c}(y|{\hat{\theta }}_{c})}$$

Assigning *y *to the class which maximises this posterior probability (the maximum probability criterion) minimises the expected misclassification error. Thus,

(3) *k *= *class*(*y*) = max{*p*(*c*|*y*)|*c *= 1,..., *C*}

To compute the posterior probabilities one needs to estimate the functions *f*_*k *_or, if the functional form is prespecified, the parameters *θ*_*k*_. The simplest functional approximation one can make is to assume that the clusters are multivariate Gaussians, so that

$$\begin{array}{l} f_{k}(y|\theta_{k})=G(y|\mu_{k},\Sigma_{k})\\ =\frac{1}{\sqrt{2\pi det\Sigma_{k}}}e^{-\frac{1}{2}{\left(y-\mu_{k}\right)}^{T}\Sigma_{k}^{-1}(y-\mu_{k})} \end{array}$$

where *μ*_*k *_is the mean and Σ_*k *_the covariance matrix of the Gaussian. If, furthermore, we assume that the covariance matrices are identical for each cluster (ie, Σ_*k *_= Σ ∀ *k*), then the classification function becomes a linear function of *y*, known as LDA. In the more general case where the covariance matrices of each class are allowed to differ, the classification function is a quadratic form of the *y *and the analysis is known as QDA.

### Mixture Discriminant Analysis

The assumption that a phenotype class is best modelled by a multivariate Gaussian is often violated. In the context of gene-expression analysis, gene expression profiles often exhibit bi-or multimodality, even when restricted to one phenotype class [5]. Similarly, gene expression profiles typically also have longer tails than Gaussians. In such circumstances, it seems more appropriate to model each *f*_*k *_as a mixture of multivariate Gaussians, since any general density can be approximated by such a mixture. Therefore, one assumes that

(4) $$f_{k}(y|\theta_{k})=\sum_{j=1}^{G_{k}}\tau_{kj}G(y|\mu_{kj},\Sigma_{kj})$$

where the number of Gaussians to use for phenotype label *k *is given by *G*_*k*_. This number may or may not be specified in advance resulting in a variety of different implementations. In ordinary MDA [22], one assumes that *G*_*k *_is known in advance for each class *k *and that the covariance matrices are all identical (ie, Σ_*kj *_= Σ). However, these assumptions are not necessary and instead one can use the training data to learn the best mixture model fit for each phenotype class using for example the Bayesian Information Criterion (BIC) [21] or a variational Bayesian framework for model selection [23]. This model selection step is a cluster-inference procedure that yields estimates for(*τ*_*kj*_,*μ*_*kj*_,Σ_*kj*_, *G*_*k*_), from which classification of test samples proceeds as before using the maximum probability criterion. Therefore, MDA is a direct generalisation of LDA and QDA and may reduce to these if the data does not support multiple components per phenotype class [21].

### Classification in heterogeneous cancers: the MDAhet classifier

Using mixtures of Gaussians, the densities of each phenotype class can be estimated more accurately. Thus, provided that the inferred Gaussian components are biologically meaningful, this approach should in general lead to an improved classification performance. However, the implicit assumption in MDA is that we are still interested in classifying samples into the *C *phenotype classes, whereas in certain circumstances we may be only interested in classifying into certain subtypes within the phenotype classes. Therefore, while in MDA one allows for heterogeneity of each phenotype label by estimating the density of each class as a mixture of Gaussians, classification is subsequently performed into each phenotype class. On the other hand, it is possible to classify samples into the Gaussian subcomponents inferred for each phenotype class, a variation of MDA called Heterogeneous Mixture Discriminant Analysis (MDAhet), because this explicitly takes the heterogeneity of each phenotype class into account by attempting to classify the samples into these subcomponents.

As an example, consider the case of two phenotype classes with MDA predicting two Gaussian components for each class. Thus, training data is used to learn the parameters and weights for four Gaussian clusters and classification of test samples is subsequently performed via the Bayes' classifier (equation 3) on these four subclasses. Note therefore that in MDAhet, the cluster-inference step of MDA is used to define the classes for which classification is then performed. Since these inferred classes make up subtypes of the original phenotype labels, this classification framework explicitly takes the heterogeneity of the phenotypes into account.

In the context of cancer gene-expression studies it has been a problem in certain cancers to derive reliable prognostic classifiers as is the case for ER- breast cancer. Typically, in the context of prognosis one would expect discriminative gene-expression profiles to exhibit bimodal distributions with the two modes mapping roughly to the two prognostic groups (good and poor) [11]. However, as previously shown [5], the best candidate gene-expression prognostic markers can also exhibit bimodal (or multimodal) profiles (ie, mixtures of Gaussians) within a given prognostic class, indicating that these phenotypes are themselves heterogeneous and that classification analysis should attempt to take this heterogeneity explicitly into account. Thus, in such circumstances the proposed classifier MDAhet seems the more appropriate classification scheme to use.

### Time-dependent negative predictive value analysis

Following the work by Heagerty and colleagues [24], we estimate time-dependent sensitivity *SE*(*t*) and specificity *SP*(*t*) values using Kaplan-Meier estimators for the predicted subclasses. In our context, we assume that samples have been classified into two groups, so that the predictor *X *= 1 predicts poor prognosis, while *X *= 0 predicts good prognosis (ie, the 'good-up' group) Thus,

$$\begin{array}{l} SE(t)=\frac{\left(1-{\hat{S}}_{KM}(t|X=1))p(X=1\right)}{1-{\hat{S}}_{KM}(t)}\\ SP(t)=\frac{{\hat{S}}_{KM}(t|X=0))p(X=0)}{{\hat{S}}_{KM}(t)} \end{array}$$

where ${\hat{S}}_{KM}$(*t*) denotes the Kaplan-Meier estimator for the overall survival function, while ${\hat{S}}_{KM}$(*t*|*X *= *c*) denotes the Kaplan-Meier survival estimate for the particular subgroup *X *= *c *(*c *= 1, 2) [24]. In our context, however, the most important performance measure is the negative prective value (NPV), since this is the probability of correctly identifying a patient with a good prognosis. Adapting the same methods as used by Heagerty and colleagues [24] we can obtain time-dependent estimates for the NPV and positive predictive value (PPV) simply as:

$$\begin{array}{l} NPV(t)={\hat{S}}_{KM}(t|X=0)\\ PPV(t)=1-{\hat{S}}_{KM}(t|X=1) \end{array}$$

## Results

### The seven-gene immune response module validates in six external cohorts

**Figure 1:**
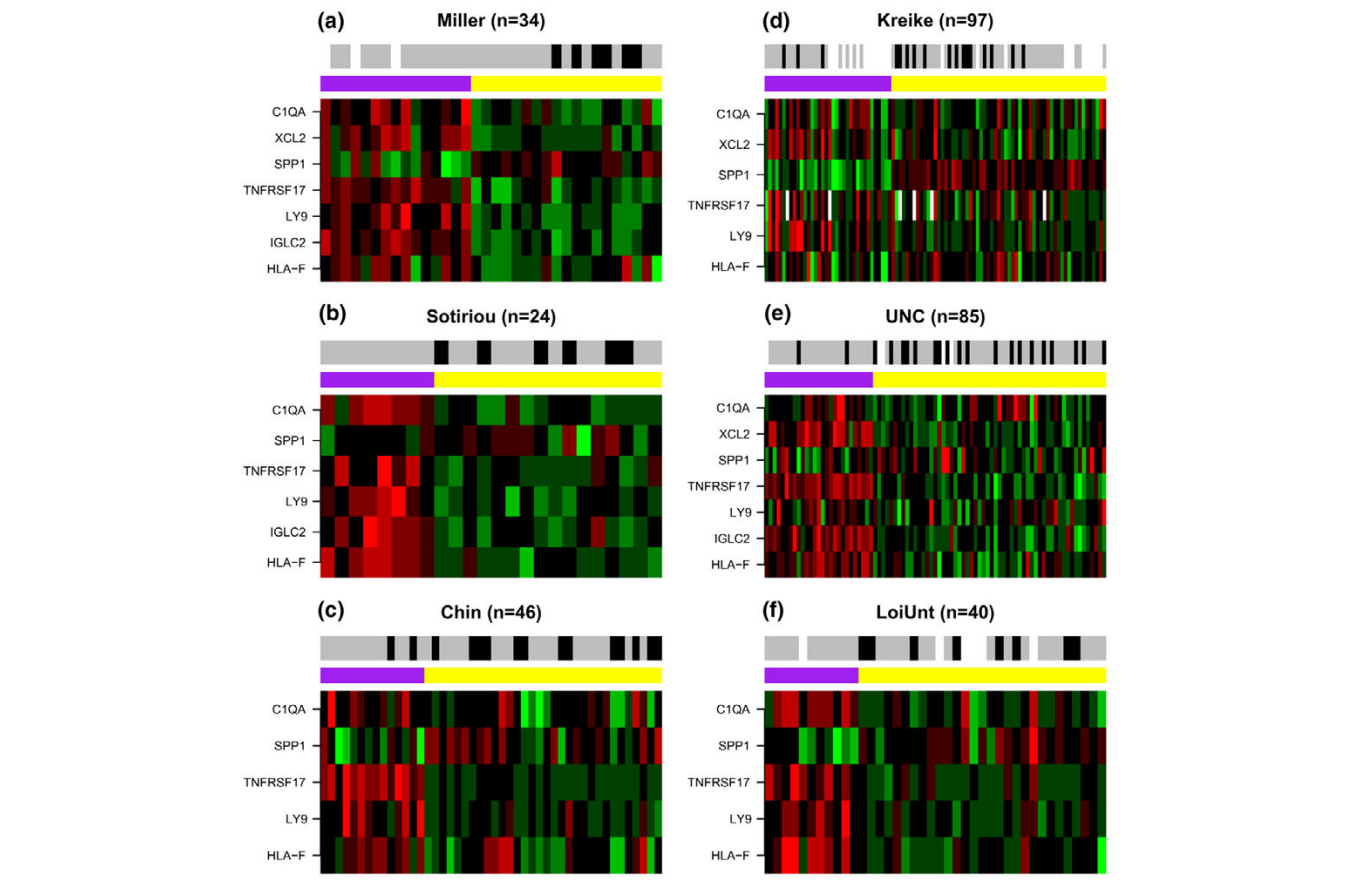
**Heatmaps of seven-gene immune response-modules**. Heatmaps of gene expression of the seven-gene immune response-module for the training and six test cohorts (red = high relative expression, green = low). Samples are clustered into two groups according to the partitioning around medoids algorithm [28] (purple = group overexpressing the immune response-module, yellow = group underexpressing the immune response-module). Clinical outcome as defined by a disease-specific death event (or distant metastasis if the former is not available) is also shown (black = poor, grey = good, white = missing data). Note that in some cases not all seven genes could be mapped to the external platform. C1QA = complement component 1, q subcomponent, A chain; HLA-F = major histocompatibility complex, class I, F; IGLC2 = immunoglobulin lambda constant 2; LY9 = lymphocyte antigen 9; TNFRSF17 = tumour necrosis factor receptor superfamily member 17; SPP1 = secreted phosphoprotein 1 (osteopontin); XCL2 = chemokine (C motif) ligand 2.

Applying a feature selection method designed to remove false positives [11] to an integrated expression data set of 186 untreated ER- samples across 5007 genes [3,8,10], we previously identified a total of 22 prognostic genes, seven of which were associated with immune response functions (*XCL2, HLA-F, C1QA, TNFRSF17, SPP1, IGLC2, LY9*) [5]. Furthermore, mapping the seven-genes into those available on two external platforms we were able to separate two independent untreated populations of ER- breast cancer patients [9,12] into two subgroups with statistically significant differences in survival outcome [5]. Specifically, samples overexpressing this module had significantly better clinical outcomes, as measured by absence of a poor outcome event (disease-specific death or the surrogate distant metastasis if the former was unavailable) (Figures 1a, b).

These results motivated us to investigate the prognostic role of the immune response-module further in four additional ER- data sets for which gene expression and clinical data were available [6,18-20]. Using the same partitioning around medoids algorithm to separate each of these additional independent cohorts into two subgroups we were able to confirm the prognostic role of the immune response-module across a total of 469 ER- tumours (Figures 1c to 1f). Given that overexpression of the immune response-module consistently identified a good prognosis subgroup of ER- breast cancer, we asked if we could derive a robust single-sample prognostic predictor.

### Deriving the prognostic classifier

**Figure 2:**
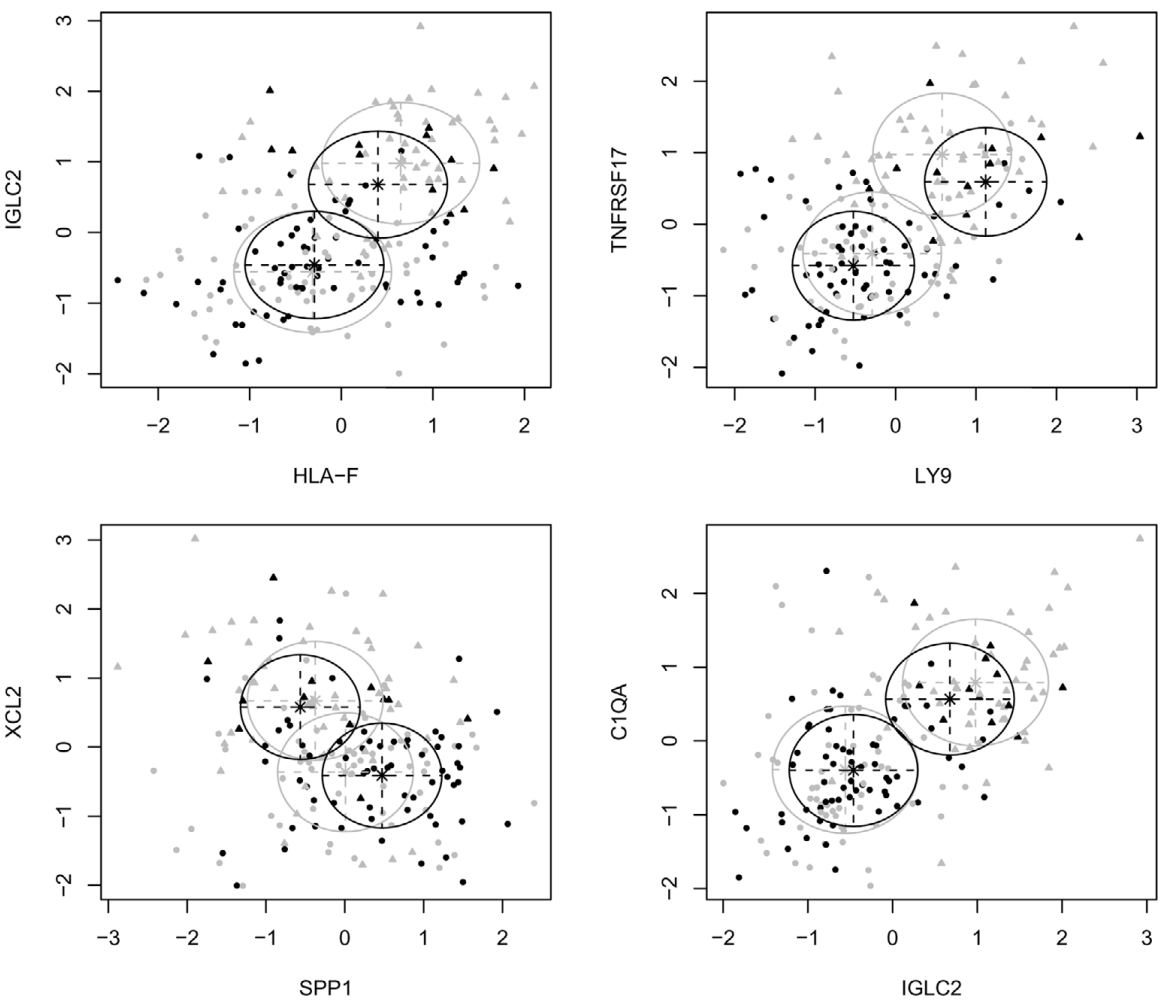
**The MDA and MDAhet classifier**. Four two-dimensional projections of the seven-dimensional Mixture Discriminant Analysis (MDA) and Heterogeneous Mixture Discriminant Analysis (MDAhet) classifiers. Scatterplots show projections of the training expression data (183 oestrogen receptor negative samples) onto arbitrarily chosen two-dimensional subspaces spanned by the genes *HLA-F *and *IGLC2*, *LY9 *and *TNFRSF17*, *SPP1 *and *XCL2*, and *IGLC2 *and *C1QA*. Codings: black = poor outcome, grey = good outcome, triangle = training samples classified into the good prognosis subgroup defined by overexpression of seven-gene module 'good-up', circle = training samples not classified into 'good-up' group. In addition, the means and covariance-curves of the two Gaussians that approximate each of the poor (black ellipses) and good outcome (grey ellipses) classes are shown. C1QA = complement component 1, q subcomponent, A chain; HLA-F = major histocompatibility complex, class I, F; IGLC2 = immunoglobulin lambda constant 2; LY9 = lymphocyte antigen 9; TNFRSF17 = tumour necrosis factor receptor superfamily member 17; SPP1 = secreted phosphoprotein 1 (osteopontin); XCL2 = chemokine (C motif) ligand 2.

To derive a single-sample prognostic classifier we first applied a mixture discriminant classifier to the same training set of 186 ER- patients and across the seven identified genes. The heterogeneity of the good-prognosis phenotype, as shown by the gene expression patterns of the immune response-module (Figure 1), suggested to us that MDA [21] would be an appropriate classification method to use, since it is designed to work for such heterogeneous phenotypes. Specifically, the MDA classifier estimates, from the training data, densities for each of the good and poor prognosis phenotypes as mixtures of two Gaussians (Figure 2). The choice of two Gaussians to model each phenotype was not arbitrary but followed from the application of a variational Bayesian algorithm that infers the optimal number of Gaussians to use [23] (data not shown). Thus, using the training data, patients with a good prognosis were divided up into two groups, one with high relative expression of the immune response-genes (the 'good-up' group) and another with relative low expression (the 'good-down' group). A similar subdivision was performed for the poor prognosis patients to yield 'poor-up' and 'poor-down' subgroups. The training process involves learning the mean expression vectors, covariance matrices and weights for each of the four subgroups (Table 1).

**Table 1:** The Heterogeneous Mixture Discriminant Analysis (MDAhet) classifier.

$\hat{\mu }$	good-down	good-up	poor-down	poor-up
HLA-F	-0.31	0.65	-0.29	0.40
IGLC2	-0.56	0.98	-0.46	0.68
LY9	-0.29	0.58	-0.52	1.12
TNFRSF17	-0.41	0.97	-0.58	0.59
SPP1	0.01	-0.38	0.47	-0.57
XCL2	-0.36	0.67	-0.41	0.58
C1QA	-0.39	0.79	-0.40	0.57
$\hat{\Sigma }$	0.74	0.74	0.58	0.58
$\hat{\pi }$ ∝ *I*	0.31	0.28	0.32	0.09

Estimated mean expression profiles $\hat{\mu }$, covariance matrices $\hat{\Sigma }$ and weights $\hat{\pi }$ for the four subgroups, as estimated from the training set. Note that the optimal covariance matrices were all proportional to the identity matrix $\hat{\Sigma }$ ∝ *I *and are thus summarised by a single value, the variance of expression of the corresponding cluster. C1QA, complement component 1, q subcomponent, A chain; HLA-F, major histocompatibility complex, class I, F; IGLC2, immunoglobulin lambda constant 2; LY9, lymphocyte antigen 9; TNFRSF17, tumour necrosis factor receptor superfamily member 17; SPP1, secreted phosphoprotein 1 (osteopontin); XCL2, chemokine (C motif) ligand 2.

### Evaluation of the prognostic classifier: MDAhet versus MDA

Having estimated the parameters for each of the phenotypes, external samples can then be classified by applying the MDA to the test sample's gene expression profile, yielding probabilities of the sample belonging to each phenotype class, and subsequently using the maximum probability criterion for class assignment. Since each phenotype class is modelled as a mixture of two Gaussians (Figure 2), class assignment can also be made on the four subclasses, a novel variation of MDA called MDAhet because this explicitly takes the heterogeneity of each phenotype in the classification process into account. This novel variation of MDA is crucial as it allows for a more reliable identification of good prognosis samples (ie, the NPV).

In detail, MDAhet assigns a test sample with a seven-gene expression profile *y *to one of the four subclasses *c *(*c *= 1, 2, 3, 4) using the maximum probability criterion

(5) $$c=\{j:\underset{j=1,2,3,4}{max}\frac{{\hat{\pi }}_{j}G(y|{\hat{\mu }}_{j},{\hat{\Sigma }}_{j})}{\sum_{k=1}^{4}{\hat{\pi }}_{k}G(y|{\hat{\mu }}_{k},{\hat{\Sigma }}_{k})}\}$$

where *G *denotes the seven-dimensional multivariate Gaussian and the parameters $\left({\hat{\mu }}_{j},{\hat{\Sigma }}_{j},{\hat{\pi }}_{j}\right)$ are estimated from the training set (Table 1).

The classification distribution of samples from the six external cohorts into the four subclasses as determined by MDAhet showed that test samples classified most often into the 'poor-down' and 'good-up' classes (Table 2). Since samples falling into the 'good-down' and 'poor-down' categories could not be discriminated in terms of prognosis (a sign that these subclasses are not distinguishable on the basis of the expression of these seven genes) we can pool these together in order to compare more objectively the predicted proportions with those estimated from the training set. This revealed that for four cohorts, JRH-2 (8 vs. 16), CAL (13 vs. 33), UNC248 (28 vs. 56) and Loi (13 vs. 27), the 'good-up' group is about half the size of the pooled 'down' group (Table 2), which is consistent with the relative proportions estimated from the training set (0.28 vs. 0.63). For the other two cohorts, relative proportions still did not deviate markedly from the training set proportions, although some deviations might be expected due to inherent cohort differences.

**Table 2:** Classification of test samples.

Test cohort	Size	good-down	good-up	poor-down	poor-up
UPP	34	4	14	16	0
JRH-2	24	5	8	11	0
CAL	46	13	13	20	0
Kreike	97	18	35	41	3
UNC248	85	28	28	28	1
Loi	40	8	13	19	0

Distribution of test samples into the four subclasses by the Heterogeneous Mixture Discriminant Analysis (MDAhet) classifier.

### Validation of MDAhet in external cohorts

To evaluate the performance of the MDAhet classifier in the training and test cohorts we used several different measures and models of prognostic separation, depending on the variable of clinical outcome used. As binary outcome we used absence or presence of a disease-specific death event, or the surrogate-distant metastasis if the former was not available. Since this does not take time dependence of events into account, binary outcome was also used at four years after surgery adapting methods for time-dependent receiver operator curve (ROC) analysis [24]. In addition, we considered continuous outcome in full stratified Cox-proportional hazard regression models, where stratification was performed on a per cohort basis to take inter-cohort differences in the types of survival data (ie, whether disease-specific survival or distant metastasis) into account.

Performance indicators based on the binary outcome measures are shown in Table 3. The most important performance indicator in our context is the NPV, since this represents the probability of correctly identifying a good prognosis patient. As shown, the NPV was very high with average values of 0.8 in the training sets and 0.96 in the test sets (range 0.85 to 1). Indeed, a significant improvement over simple predictions based on *a priori *known proportions was observed in all test sets (Table 3). In line with these results, sensitivity values were also very high with average values of 0.84 in training sets and 0.94 in test sets (range 0.76 to 1). Results evaluated at four years after surgery were, as expected, not markedly different, indicating that the prognostic classifier performs equally well in terms of short-term survival outcomes (Table 3).

**Table 3:** Performance measures of seven-gene Heterogeneous Mixture Discriminant Analysis (MDAhet) classifier

	Training set			Test	Sets		
Cohort	NKI2+EMC+NCH	UPP	JRH-2	CAL	Kreike	UNC248	Loi

Cohort size	186	34	24	46	97	85	40
Annotated	183	31	24	46	71	80	34
Good prognosis (%)	59	81	75	67	76	74	76
Poor prognosis (%)	41	19	25	33	24	26	24
Chemotherapy (%)	0	0	0	67	0	66	0
MDA							
NPV (%)	74	92	93	69	83	74	100
PPV (%)	55	28	56	35	29	27	40
SE (%)	69	83	83	53	71	38	100
SP (%)	61	48	78	52	44	63	54
MDAhet							
**NPV **(%)	**80**	**100**	**100**	**100**	**85**	**92**	**100**
PPV (%)	51	30	37	45	29	36	35
**SE **(%)	**84**	**100**	**100**	**100**	**76**	**90**	**100**
SP (%)	44	44	44	42	41	42	42
**NPV at 4 years **(%)	**83**	**100**	**100**	**100**	**88**	**93**	**100**
PPV at 4 years (%)	42	24	33	35	25	45	35
**SE at 4 years **(%)	**83**	**100**	**100**	**100**	**79**	**88**	**100**
SP at 4 years (%)	44	42	43	37	40	45	43
LN							
NPV (%)	61	84	NA	85	NA	85	76
PPV (%)	50	30	NA	46	NA	37	0^a^
SE (%)	27	50	NA	80	NA	71	0^a^
SP (%)	81	70	NA	55	NA	58	100^a^
NPV at 4 years (%)	67	88	NA	90	NA	82	77
PPV at 4 years (%)	39	37	NA	38	NA	47	0^a^
SE at 4 years (%)	25	84	NA	85	NA	69	0^a^
SP at 4 years (%)	80	74	NA	53	NA	60	100^a^

^a^Loi's cohort consists only of LN- samples. The table summarises performance indicators of the seven-gene MDAhet classifier and lymph node status (LN) across oestrogen receptor negative (ER-) training and test sets. For each cohort, we also give the number of tumours (cohort size), number of clinically annotated tumours (annotated), the percentage of good and poor prognosis patients (as defined by disease-specific death or distant metastasis event) and the percentage of patients treated with chemotherapy. NPV, PPV, SE and SP are evaluated at four years and at end of study. NPV, negative predictive value (precision for good prognosis); PPV, positive predictive value (precision for poor prognosis); SE, sensitivity; SP, specificity.

Stratified Cox-regression models further confirmed the much better prognosis of the predicted subclass overexpressing the immune response-module relative to samples classified as poor prognosis (Table 4). Specifically, samples classified as good prognosis with overexpression of the immune response-module ('good-up' group) have less than half the risk of a poor outcome event (death or distant metastasis) relative to samples classified as poor prognosis, a result that we found to be independent of LN status and chemotherapy (Table 4). Note that four of the test cohorts were untreated (no chemotherapy) populations (Table 3), such as the training set itself, confirming the prognostic relevance of the classifier, and that chemotherapy itself was not prognostic in the two partially treated populations (Table 4).

**Table 4:** Stratified Cox-regression model of seven-gene Heterogeneous Mixture Discriminant Analysis (MDAhet) classifier

	Training set	Combined test set
Annotated	183	286
MDAhet	0.29 (0.16–0.56) *p *= 0.0002	0.15 (0.07–0.36) *p *< 0.000001
LN	1.31 (0.73–2.33) *p *= 0.36	3.25 (1.61–6.58) *p *= 0.001
CT	NA	0.68 (0.34–1.39) *p *= 0.29
LN+MDAhet		
MDAhet	0.29 (0.15–0.55) *p *= 0.0002	0.06 (0.01–0.27) *p *= 0.0002
LN	1.59 (0.81–3.11) *p *= 0.18	3.68 (1.32–10.13) *p *= 0.012
CT+MDAhet		
MDAhet	NA	0.27 (0.15–0.48) *p *= 0.00001
CT	NA	0.76 (0.27–2.13) *p *= 0.6

Stratified Cox-proportional hazards regression performance of the seven-gene MDAhet classifier, lymph node status (LN) and chemotherapy (CT) across oestrogen receptor-negative training and test sets, with strata defined by cohorts. For the univariate analysis, Hazard ratio (HR), 95% confidence intervals (CI) and LR-test p-value are given. In the multivariate models, p-values quoted are from the corresponding Wald test.

**Figure 3:**
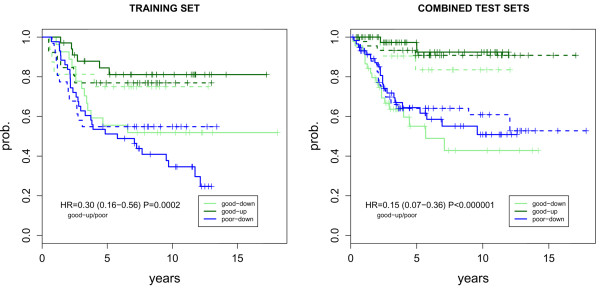
**Kaplan-Meier curves for MDAhet classifier**. Kaplan-Meier survival curves for the three subclasses 'good-down' (light green), 'good-up' (dark green), 'poor-down' (blue), as predicted by the Heterogeneous Mixture Discriminant Analysis (MDAhet) classifier, in the training and combined test cohorts. The class 'poor-up' is not shown due to small sample size (Table 2). Hazard ratios (HR), 95% confience intervals (CI) and log-rank test p-values are given for the predicted 'good-up' class relative to the predicted poor prognostic classes, as given by a stratified Cox-regression model with strata defined by cohorts. The Kaplan-Meier curves for each subclass is shown separately for disease-specific survival (solid lines) and distant metastasis (broken lines).

Kaplan-Meier survival curves stratified according to the type of survival data (disease-specific death or distant metastasis) further confirmed the better prognosis of the predicted 'good-up' group (Figure 3). These survival curves further show that the classifier in the test sets is unable to discriminate the good prognosis samples that do not overexpress the immune response-module ('good-down') from the poor outcome samples. This result is expected since the seven-gene module is hypothesised to only identify a particular subgroup of good prognosis [5].

Since the maximum probability criterion assigns test samples to classes without regard to how large the maximal posterior class probabilites are, we tested the robustness of our results by only classifying samples passing a minimum probability threshold. For a probability threshold of 0.3 (already significant compared with the minimum possible maximal probability of 1/4 = 0.25), 94% of all test samples passed this threshold, indicating that our results are indeed robust. For a threshold of 0.4, we found 68%of samples were classifiable and results were still in line with those reported for the minimum threshold of 0.25 (data not shown).

## Discussion

Based on the seven genes we had identified previously as defining an immune response-related prognostic module in ER- breast cancer, we have now constructed a single-sample classifier and have validated it in six external, independent ER- cohorts, four of which were untreated populations. Remarkably, we find that overexpression of this immune response-module considerably reduces the risk of disease-specific death or distant metastasis in both untreated and partially untreated ER- populations (HR = 0.15; 95% confidence interval 0.07 to 0.36; *p *< 10^-6^) (Table 4). Importantly, we also found that this association is independent of LN status (Table 4). In terms of binary outcome measures, the classifier shows clinical promise with consistently high NPV values across all test cohorts, even when time-dependent outcome measures are taken into account (Table 3). For example, the NPV and sensitivity values at four years after surgery were 100% in four of the six cohorts and in all cases larger than 85%. Thus, the classifier could potentially be used for identifying high-grade ER- patients that may benefit from a less agressive or nonexistent course of chemotherapy.

The remarkably high NPV values in the test cohorts, however, raise some important questions. First, we found that the performance in the test sets was better than in the training set (Tables 3 and 4). While this is true for the NPV analysis, the Cox-regression analysis also shows that the 95% confidence intervals (CI) are overlapping. Therefore, statistically, there is no discrepancy. In any case, a plausible explanation for why the performance is slightly worse in the training set could be related to the merging step involved in building the training set [5]. By merging different microarray expression sets together we gain power from the considerable increase in sample size; however, merging may also compromise the accuracy of the expression profiles, because these need to be renormalised before merging is performed [5]. Therefore, it is entirely plausible that small errors in the merging procedure may have affected the classifier's performance in the training set. In this context it is important to point out that the training set is only used to derive a classifier and that the gold-standard evaluation of any classifier is determined by its performance in the test cohorts [25]. As shown here, the MDAhet classifier is strongly prognostic across six totally independent breast cancer cohorts profiled on different array platforms.

A second important point relates to the nature of the MDAhet classifier. As remarked in a previous study [9], in the context of validating gene expression signatures across different array platforms, some renormalisation is inevitable. Thus, our MDAhet classifier is not strictly speaking a single-sample predictor because the gene expression value of a test sample needs to be renormalised (a simple centering and scaling) across all the test samples in the same cohort, before classification is performed. However, this does not preclude the classifier from being a potential single-sample predictor because in the clinical setting such platform differences would not exist and so no normalisation step would be necessary. Hence, in line with other classifiers presented in the literature [9,26] our MDAhet classifier is also a single-sample predictor because, modulo the normalisation step, the classification is performed solely with information taken from the training set (Table 1).

Given the association of overexpressed immune response related genes with good prognosis in ER- breast cancer, as supported now by several studies [5,13-16], it is natural to ask about the biological meaning of such overexpression. One plausible explanation for the overexpression of immune response genes in these tumours is a higher degree of LI, because some of the genes involved are lymphocyte markers [13-15] and LI itself is associated with good prognosis in ER- breast cancer patients [6,14,15]. However, there is also evidence for a more complex role of the mRNA expression of these genes [5]. First, it was found that the prognostic performance of the seven-gene module previously reported [5] was independent of LI. Second, it was shown that the good prognosis class was heterogeneous with only about half of the cases mapping closely to medullary breast cancer, a morphologically distinct subclass associated with high LI and marginally better prognosis as compared with the other ER- subtypes (ie, the basal and the HER2+ subtypes) [5,27]. Thus, the best prognosis is attained by the other half of the samples that are not necessarily related to high LI and medullary breast cancer [5]. All these findings are consistent with the marginal association of LI or LI-associated gene expression with good prognosis in ER- breast cancer, as reported recently [6,13-15], and suggest that only part of the overexpression of the immune response-module is due to LI [5]. Lending further support to this, it was also found that one gene member (*SPP1*) is consistently underexpressed in patients with a good prognosis. To conclude, we can therefore hypothesise that the MDAhet classifier and associated immune response-module may be identifying another good prognosis ER- subset of tumours, but with a significantly better prognosis than medullary high-LI breast cancer (Tables 3 and 4). In any case, even if the expression pattern of the immune response-module is entirely due to variable LI, the MDAhet classifier appears to provide a much more reliable prognostic classifier than LI-scores derived from immunohistochemistry [6] or lymphocyte-specific gene expression markers [14,15]. Further larger studies with reliable LI data are required to answer these questions conclusively [15].

## Conclusion

We have derived a single-sample classifier for good prognosis in ER- breast cancer with a high predictive value (in test sets, mean NPV = 94%, range 85 to 100%) in six independent test cohorts and validity in more than 469 patients, and which performs independently of LN status. We propose to develop a reverse transcriptase-polymerase chain reaction-based clinical assay based on these seven genes to identify ER- patients of good prognosis that may benefit from a less aggressive course of chemotherapy.

## Abbreviations

C1QA: complement component 1, q subcomponent, A chain; CI: confidence intervals; CT: chemotherapy; ER: oestrogen receptor; HER2: human epidermal growth factor receptor 2; HLA-F: major histocompatibility complex, class I, F; HR: hazard ratio; IGLC2: immunoglobulin lambda constant 2; LDA: Linear Discriminant Analysis; LI: lymphocytic infiltration; LN: lymph node; LY9: lymphocyte antigen 9; MDA: Mixture Discriminant Analysis; MDAhet: Heterogeneous Mixture Discriminant Analysis; NPV: negative predictive value; PPV: positive predictive value; QDA: Quadratic Discriminant Analysis; ROC: receiver operator curve; SPP1: secreted phosphoprotein 1 (osteopontin); TNFRSF17: tumour necrosis factor receptor superfamily member 17; XCL2: chemokine (C motif) ligand 2.

## Competing interests

The authors declare that they have no competing interests.

## Authors' contributions

AET conceived of the study, performed all statistical analyses and wrote the manuscript. CC contributed to the writing of the manuscript.
